# Targeting Senescence as a Therapeutic Opportunity for Triple-Negative Breast Cancer

**DOI:** 10.1158/1535-7163.MCT-22-0643

**Published:** 2023-02-07

**Authors:** Bruno de Paula, Rosalind Kieran, Samantha Shui Yuan Koh, Susanne Crocamo, Eliana Abdelhay, Daniel Muñoz-Espín

**Affiliations:** 1 Breast Cancer Research Unit, Instituto Nacional de Cancer, Rio de Janeiro, Brazil.; 2 Early Cancer Institute, Department of Oncology, Cambridge University Hospitals Foundation Trust, Cambridge, United Kingdom.; 3 Department of Medicine, Cambridge University Hospitals Foundation Trust, Cambridge, United Kingdom.; 4 Instituto Nacional de Cancer, Rio de Janeiro, Brazil.

## Abstract

Triple-negative breast cancer (TNBC) is associated with an elevated risk of recurrence and poor prognosis. Historically, only chemotherapy was available as systemic treatment, but immunotherapy and targeted therapies currently offer prolonged benefits. TNBC is a group of diseases with heterogeneous treatment sensitivity, and resistance is inevitable and early for a large proportion of the intrinsic subtypes. Although senescence induction by anticancer therapy offers an immediate favorable clinical outcome once the rate of tumor progression reduces, these cells are commonly dysfunctional and metabolically active, culminating in treatment-resistant repopulation associated with worse prognosis. This heterogeneous response can also occur without therapeutic pressure in response to damage or oncogenic stress, playing a relevant role in the carcinogenesis. Remarkably, there is preclinical and exploratory clinical evidence to support a relevant role of senescence in treatment resistance. Therefore, targeting senescent cells has been a scientific effort in many malignant tumors using a variety of targets and strategies, including increasing proapoptotic and decreasing antiapoptotic stimuli. Despite promising results, there are some challenges to applying this technology, including the best schedule of combination, assessment of senescence, specific vulnerabilities, and the best clinical scenarios. This review provides an overview of senescence in TNBC with a focus on future-proofing senotherapy strategies.

## Introduction

Breast cancer is the most common cancer among women in the United Kingdom, accounting for 15% of all new cancer cases, and it is the second most deadly cancer after lung cancer (1). Gene expression studies have shown that it can be subdivided into intrinsic molecular subtypes. The main ones are Luminal A, Luminal B, and HER2-enriched and basal-like (triple-negative) breast cancer, and each has significant differences in incidence, risk factors, prognosis, and treatment sensitivity (2). Compared with the Luminal A and Luminal B subtypes, basal-like triple-negative breast cancer (TNBC) is markedly distinct.

Most TNBC can only be treated with cytotoxic chemotherapies such as epirubicin, cyclophosphamide, docetaxel, and doxorubicin, among others, whereas ER/PR positive subtypes respond to anti-estrogens and HER2 enriched to monoclonal humanized antibodies alone or in addition to these chemotherapy regimens. TNBC, on the other hand, shows a far more aggressive nature, and early response to these chemotherapy regimens is often rapidly followed by resistance and relapse. Other agents such as PARP inhibitors, whose effects are mediated via DNA damage repair pathway (DDR) and the checkpoint inhibitors, have been added to the armory for the treatment of BRCA1/2 mutated disease (3, 4). However, they have not shown the same success as the anti-hormone and HER2 treatments for the other subtypes. Despite comprising just 15% of all breast cancers, TNBC accounts for 24% of breast cancer mortality, and >50% of patients with metastatic disease die within 1 year of diagnosis (5). Studies are increasingly showing that cellular senescence plays an important role in the propensity for TNBC to show resistance to treatments such as chemotherapy, immunotherapy (6), and treatments targeting the DNA damage response (DDR; ref. 7) and early relapse.

Cellular senescence is widely accepted as a highly complex and multifaceted process playing physiologic and reparative roles necessary for normal tissue homeostasis and remodeling, such as during embryogenesis, wound healing, host immunity, and tumor suppression (8). These processes can be triggered by a number of stimuli including, among others, developmental programmes, telomere shortening (replicative senescence), genotoxic stress in response to treatment (therapy-induced senescence, TIS), oncogene activation (oncogene-induced senescence, OIS), cellular damage or stress (damage-induced senescence), mitochondrial dysfunction-associated senescence, and paracrine senescence (9). Because senescence facilitates the removal of ages, oncogenically-activated or damaged cells akin to apoptosis, it is particularly relevant in preventing cancer progression. This process usually implies the removal or clearance of senescent cells by the immune system (10).

Conversely, when the process of immunosurveillance and clearance deregulates, senescent cells can persist in tissues, leading to a variety of cell autonomous and non-cell autonomous tumor-promoting activities, as described in more detail in this review. Concerning breast cancer, normal mammary gland development and function relies on regular monthly cycling of secretion of estrogen and progesterone, which act on their respective receptors leading to mammary gland epithelial cell and stromal proliferation and remodeling (11). These processes result in cellular stress and DNA damage, which have the potential of initiating steps in neoplastic transformation on the one hand, and protective senescence, which allows for healing after tissue damage on the other. Accordingly, senescence has been found in early breast cancer lesions and promote tumor growth (12).

Initially described by Hayflick and Moorhead in 1961 in fibroblast cultures (13), cellular senescence limits cells’ capacity to proliferate via three broad stages: first, cells enter a stable nonproliferating stage, followed by clearance via phagocytosis; and then remodeling (14). Entrance into a senescent stage is mainly implemented via inhibition of the cyclin-dependent kinase pathways and ultimately the p53/p21 and p16/Rb pathways resulting in stable cell-cycle arrest (15). Physiologic clearance of senescent cells depends on DNA damage resulting in the particular cocktail of cytokines, chemokines, proteases, and growth factors, which facilitates immune clearance by recruiting NK cells, macrophages, and neutrophils (16). However, immune clearance can be inhibited, allowing senescent cells to persist with chronic—deregulated—senescence-associated secretory phenotype (SASP) production. The SASP is highly inflammatory and can lead to malignant transformation of neighboring cells via paracrine effects (10). This is of relevance to the treatment of TNBC in the context of DNA damage caused by cytotoxic drugs used in TNBC.

TNBC often results in a more rapid initial response to chemotherapy than other breast cancer subtypes. Chemotherapy causes DNA damage inducing apoptosis and senescence in malignant cells; this is followed by immune clearance of these cells and manifests as a rapid disease response (17, 18). However, eliminating immune-sensitive cells results in a selective pressure that favors the persistence of immune-resistant subclones, which continue to divide (competitive release) and propagate malignant effects (19).

As mentioned, PARP inhibitors have been approved to treat BRCA1/2-mutated breast cancer (3). They cause the accumulation of single-stranded breaks (SSB) in DNA, which, when used in conjunction with nonfunctioning BRCA1/2 genes, leads to “synthetic lethality” (whereby cells with perturbations in one gene are viable, whereas perturbations in 2 genes results in cell death) and tumor regression. Studies have shown that PARP inhibition leads to p53-independent cellular senescence with SASP production, potentially implicating TIS as a resistance mechanism to treatment with PARP inhibitors, akin to resistance to chemotherapy via TIS. Furthermore, PARP inhibitor-induced senescent cells begin to proliferate on withdrawal of the PARP inhibitor, indicating the need for sustained PARP inhibitor use (7).

Similarly, initially, OIS is a cellular mechanism that protects against the malignant transformation of early neoplastic cells. For example, driver mutations in *Ras, BRAF, AKT, MYC, E2F1, Cyclin E, HER2/neu*, or in mutations in tumor suppressor genes such as *TP53, PTEN, BRCA1/2, INK4A*, and *NF1*, can lead to the senescent programme. Furthermore, the immunosurveillance of senescent cells has been fundamentally reported in liver cancer models (20, 21). However, although the SASP promotes immune clearing, paradoxically, it can have tumor-promoting effects. For instance, VEGF and IL6, which are secreted in the SASP, can contribute to angiogenesis and invasiveness (22), which are hallmarks of tumorigenesis. A further example is that, although MCP1 and CXCL promote clearance of senescent cells by recruitment of NK cells and macrophages, MCP1 can also increase immune resistance and reduce the clearance of senescent cells via its recruitment of immature myeloid cells and M2 macrophages (23).

In recent years, novel therapeutic strategies have focused on targeting and eliminating senescent cells by using pharmacologically-active compounds (senolytics) to prevent tumorigenic effects. Ideally, such senotherapies would target markers or hallmarks of senescence. There are several markers that characterize senescence, such as senescence-associated β-galactosidase (SA-β-gal) activity, high p16 protein expression, trimethylated histone H3 lysine 9 (H3K9me3), absence of markers of proliferation (given that stable cell-cycle arrest is a hallmark of cellular senescence) such as Ki67 protein or 5-bromodeoxyuridine (BrdU) incorporation, or the upregulation of prosurvival pathways amongst others (10). Other classes of senotherapies are senomorphic or senostatic drugs, which target or manipulate the SASP. Another line of chemotherapeutic focuses on an intrinsic metabolic vulnerability of SASP-producing senescent cells. The SASP produces high levels of proteotoxic stress requiring high lysosomal activity and autophagy levels. Therefore, targeting lysosomal ATPases has been shown *in vitro* and *in vivo* to lead to caspase-12- and caspase-3-mediated endoplasmic-reticulum-related apoptosis and, therefore, elimination of senescent cells by exploiting this metabolic vulnerability (24).

There are several ongoing studies that are investigating the targeting of senescent cells. But they are not lacking challenges, given that cellular senescence plays a normal physiologic role, which must not be upset by attempts to target senescent malignant cells.

The next sections describe the enriched pathways and genes involved in cellular senescence in TNBC, potential therapeutic strategies, and the challenges faced by targeting cellular senescence as a safe and effective anticancer therapy.

## Triple Negative and Senescence

### Enriched pathways and genes

TNBC is not a homogeneous subtype of breast cancer. It represents a group of multiple disease spectrums with different biologic behavior (25). Lehmann and colleagues (26) reported that across several triple-negative cell lines, six intrinsic subtypes are associated with different genomic features and canonical pathways.

Core pathways and genes include basal-like 1 (BL-1) cell cycle and DNA repair genes pathways *ATR/BRCA, MYC*; proliferation genes such as *AURKA* and *B*; basal-like 2 (BL-2) growth factor pathways including *EGF, MET*, and glycolysis/glycogenesis, the immunomodulatory (IM) immune cell signaling, TNF and JAK, mesenchymal-like (M) and mesenchymal stem-cell-like (MSL) IGF/mTOR, NOTCH1, and ALK pathways; enrichment of *BCL2* genes; and finally the luminal androgen receptor (LAR) enriched pathways of androgen and estrogen metabolism (26).

More specifically, aurora kinase A (AURKA) belongs to the serine/threonine kinases, the TGCA has shown are more highly expressed in cancer cells than in normal control cells. KEGG pathway enrichment and GO analysis have shown that AURKA interacts with proteins involved in cellular processes such as mitosis, cell-cycle progression, and apoptosis, as well as crucial oncogenic pathways such as the PI3K/AKT, mTOR, β-catenin/Wnt, and NF-κB (27).

Proto-oncogene tyrosine-protein kinase Src proteins are nonreceptor tyrosine kinases (SFKS) implicated in cellular processes such as proliferation, migration, differentiation, and survival. Dysfunction of this proto-oncogene Src is therefore associated with tumorigenesis. It also plays an essential role in the activation of JAK2/Stat 5 upon prolactin receptor stimulation, which has been shown to trigger a pattern of gene expression that contributes to mammary development and tumorigenesis (28).

TP53 is mutated in approximately 80% of TNBC and other difficult-to-treat cancers. It is a tumor suppressor gene that acts as a homotetrameric transcription factor, binding to specific DNA sequences regulating cell-cycle arrest, apoptosis, or senescence, and modulating other cellular processes, including metabolism, stem-cell maintenance, invasion, and metastasis. Generally, Tp53 mutations occur within the DNA-binding domains (DBD), which interfere with DNA binding. In addition to loss of wild-type p53 tumor suppressor function, tumorigenesis can occur via gain of function TP53 interactions with other transcription factors, such as nuclear factor Y, vitamin D receptor, p63, and p73, which promote increased survival, proliferation, migration, and invasion (29).

Compared with other breast cancer subtypes, EGFR is overexpressed in TNBC (30). EGFR has multiple downstream effects on cell-cycle progression, transcription, and proliferation (which are deregulated in tumorigenesis) via various pathways such as MAPkinase, PI3K/PTEN, JAK/STAT pathways, among others. Furthermore, deregulated EGFR signaling has been implicated in therapeutic resistance of TNBC to chemotherapy drugs such as doxorubicin, daunorubicin, paclitaxel, cisplatin, and 5-FU (31). Downstream of EGFR, gain of function mutations in the PI3K/AKT/mTOR pathway (associated with cell metabolism, proliferation, and differentiation) are commonly found in TNBC. Most frequently, oncogenic mutations occur in the E545 helical and H1046 kinase domains of the PIK3CA gene. This has led to the development of PI3K and AKT inhibitors (32). B-cell lymphoma 2 (BCL-2) is a pro-apoptotic protein with four domains BH1, BH2, BH3, and BH4. Impaired apoptosis is a vital step in tumorigenesis. Its effects are mediated via DNA damage/p53 pathway, survival/NF-κB pathway, estrogen pathway, STAT pathway, and PI3K/Akt pathways (33).

Although several senescence inducers to TNBC will be discussed in the following sections, the phenotypes resulting from distinct compounds might differ. In a proteomic characterization of senescent MDA-MB-231 cells induced by Palbociclib, there were significant cyclin expression changes, immune response activation, upregulation of metabolic pathways, and possibly epigenetic changes (34). In another study evaluating extracellular vesicles from paclitaxel-induced Cal51 senescent cells, an increased abundance of annexins, ATP-dependent integrins, tubulins, insoluble SASP factors, and Rab proteins were found (35). Upregulation of adhesion proteins and lipid metabolism was reported by both studies and some proteins such as KRT8. Overall, a different proteasome is observed between the two studies.

Cellular senescence plays an important role in benign and malignant tissue, especially as a treatment resistance mechanism, but the specific role in TNBC carcinogenesis remains unclear. It is known that MCF10A cell lines can escape senescence upon HRAS overexpression, suggesting that senescence might play a relevant role in tissue transformation (36). On the other hand, a study evaluating the stroma of chemotherapy-naïve older patients with TNBC showed, among other senescent-related genes, the presence of SASP. These suggest that senescent cells, even with the secretory phenotype, can be found spontaneously before treatment rather than necessarily require treatment pressure to appear. Moreover, different upregulated genes were found between young and old stromal samples. The young stroma shows genes involved in stabilizing the extracellular matrix and stimulating differentiation, precluding migration and invasion, whereas the old matrix shows proliferation de-differentiation and angiogenesis-involved genes (37).

Noncoding and coding RNAs are known regulators of cell senescence. For instance, in an experiment using MDA-MB-231cell-lines treated with cisplatin and deuterium-depleted water (DDW) as senescence inducer/adjuvant treatment, the authors reported a significant downregulation of apoptosis, autophagy, senescence, drug resistance, and mitochondrial metabolism-related, compared with upregulation in cells treated with DDW versus cells maintained in standard conditions. Some target genes corresponding to the downregulated mRNAs were *PI3k/AKT, PTEN*, and those corresponding to the upregulated mRNA were *K-Ras, VEGF-A*, and *STAT3* (38).

Therefore, knowing the characteristics of each intrinsic subtype of TNBC is crucial to designing preclinical and clinical studies once specific biomarkers of sensitivity to targeted agents could optimize the drug development (39–41).

### Inducing senescence in TNBC

Many anticancer agents have been shown to induce senescence in breast cancer and are summarized in Table 1. Chemotherapy drugs, the main and only active treatment for TNBC for decades, are the most frequent TIS of choice to evaluate senolytics candidates by the one-two punch strategy (TIS followed by senolytics approach; ref. 19). The preclinical studies using this strategy in TNBC are summarized in Table 2.

**Table 1.** Examples of senescence inducers in TNBC.

Anticancer agent	Prosenescence	Model	Main effects
**Chemotherapy**	Adriamycin (141)	MDA-MB-231	Induces senescence and represses telomerase activity; in mutant p53 cells causes delayed apoptosis rather than senescence
	Doxorubicin, irinotecan, methotrexate, 5-fluorouraci, oxaliplatin, or paclitaxel (42)	MDA-MB-231 cell lines	Irinotecan and doxorubicin were more potent inducers than other agents and higher induction of γ-H2AX expression in senescent cells. Paclitaxel, 5-FU, and oxaliplatin did not increased SA-β-gal-positivity significantly
	Doxorubicin (43)	MDA-MB-231	Senescent cells showed increased sensitivity to T cells
	Paclitaxel (35)	Cal51 cell lines	Senescent cells displayed an increased expression of the multidrug resistance protein 1/p-glycoprotein
	Vinorelbine ± 5-fluorouracil (45)	MDA-MB-231 cell lines	Senescence induction is dose and schedule dependent
**Targeted therapy and/or radiotherapy**	Olaparib (7)	MDA-MB-231 cell lines	Gene-expression analysis demonstrated that, among other genes, BCL-XL was significantly upregulated following olaparib
	Radiotherapy (142)	MDA-MB-231	Radiotherapy induces senescence and telomere disfunction in p53 wild type. Accelerated senescence is p53 dependent
	Radiation + veliparib (143)	4T1 cell lines	Re-engineered cancer cells to express PD-L1 responded to immunotherapy
**CDK4/6 inhibitors**	Palbociclib (47)	4T1 cell lines	Significant increase on Bcl-xL protein expression
	Palbociclib (144)	MCF-7, MDA-MB-231 cells	CDK4/6 stabilizes and activates FOXM1, protecting cancer cells from senescence
	Abemaciclib (48)	MDA-MB-453 + xenografts	Beyond inducing senescence also promotes antitumor immunity
	Abemaciclib (34)	MDA-MB-453	D-type cyclins are activated by genomic aberrations and enhance sensitivity to CDK4/6 inhibitors
**BET inhibitors**	JQ1, I-BET151, and I-BET762 (113)	MDA-MB-231 cells	Senescence induction and associated with MED1 and hyper-phosphorylation of BRD4 attributable to decreased activity of PP2A

Abbreviations: 5-FU, 5-fluorouracil; CDK4/6, cyclin-dependent kinase 4/6; PD-L1, programmed death-ligand 1.

**Table 2.** Examples of “one-two punch” strategy in TNBC.

Prosenescence agent and reference	Senolytic	Model	Main effect
Doxorubicin (145)	Navitoclax (ABT-263)	MMTV-PyMT implanted *in vivo*	ABT-263 eliminated senescent cells induced by doxorubicin. Delayed tumor recurrence was observed when doxorubicin + ABT 263 were given postsurgical removals of tumors from MMTV-PyMT–injected cells
Doxorubicin or radiotherapy (146)	ABT-263, ABT-199, a BCL2 inhibitor, A-1331852, a BCL-XL inhibitor, and S63845, an MCL1 inhibitor	Cal51	Cal51 cell death was observed when exposed to a combination of A-1331852 or ABT-263, BCL-XL inhibitors, and S63845, an MCL1 inhibitor, but not to ABT-199, a BCL2 inhibitor; BCL-XL and MCL1 mediate survival in senescent cells
Doxorubicin or etoposide or radiotherapy (147)	Navitoclax	MDA-MB-231 cells and xenografts	Apoptosis following ATB-263 was observed in senescent cells induced by prosenescent agents. Prolonged tumor suppression following ABT-263 was observed with etoposide or doxorubicin induction
Palbociclib (133)	Navitoclax and galacto-navitoclax	4T1 implanted in mouse	Senescence clearance was observed in response to the encapsulated drug and led to less toxicity
Palbociclib (47)		4T1 implanted in mouse	
Etoposide (55)	Quercetin and derivates	MDA-MB-231 cells	Induced cells to apoptotic death and decreased responses related to pro-inflammatory and HSP70.
Radiotherapy (148)	Dasatinib + quercetin	B16F10 (melanoma cell lines) injected in mice	Radiotherapy + combination of drugs induced cell death time dependent
JQ1 (117)	Navitoclax	MDA-MB-231, HCC38, BT549, HCC1143, HCC70, and MDA-MB-468	BCL-XL inhibition induces apoptosis in BETi-induced senescent cells
Alisertib or etoposide (97)	ABT-263	MDA-MB-231 cells	MMP1, IL6, SAA1, and SAA2 are highly expressed on senescent cells as parental cells
Olaparib (7)	ABT-263, A-1155463 and piperlongumine	MDA-MB-231 cells	Upregulation of Bcl-XL after olaparib

#### Chemotherapy

Using MDA-MB-231 cell lines, Bojko and colleagues demonstrated induction of senescence following treatment with Doxorubicin or Paclitaxel. The cells were treated with the drug for 24 hours and were analyzed for senescent markers 3 days after drug removal. The senescent markers that were looked at included SA-β-gal positivity, cell granularity, levels of cytokines (IL6, IL8, VEGF), and 53BP1 and γH2AX foci. 100 nmol/L Doxorubicin and 28 nmol/L of Paclitaxel were used. Cells treated with doxorubicin had characteristic senescence morphologic changes, increased cellular granularity, and elevated SA-β-gal activity. Paclitaxel led to elevated expression of p21 and γH2AX, increased granularity of cells, and secretion of VEGF; however, there was no significant increase in SA- β-gal positive cells (42).

Inao and colleagues similarly demonstrated induction of senescence in MDA-MB-231 and BT-549 cell lines after treatment with 250 and 100 nmol/L doxorubicin, respectively. The cells showed positive SA-β-gal staining and γH2AX expression. XDR treatment leads to increased expression of p21 in MDA-MB-231 and p16 in BT-549. Furthermore, both cell lines produced higher levels of IL6 and IL8 than untreated cells (43).

Di and colleagues showed that doxorubicin could lead to bystander effects. Thus MCF-1 cells not treated with Doxorubicin were grown with MCF-1 cells, which had undergone senescence after treatment with Doxorubicin. This led to the naïve MCF-1 cells displaying features of senescence. However, this was seen in MCF-1 cells, which are not triple negative (44).

Kavanagh and colleagues used Paclitaxel to induce senescence to investigate extracellular vesicles released from therapeutic-induced senescent (TIS) TNBC cells. Cal51 TNBC cells were treated with 75 nmol/L Paclitaxel for 7 days, after which confirmation of therapeutic induced senescence was carried out through positive SA-β-gal staining, negative Ki67 staining, overexpression of p16 and p21 on Western blot analysis, as well as evidence of G_2_–M cell-cycle arrest. The authors found that TIS cells released more extracellular vesicles than the control and hypothesized that the release of these vesicles, many of which contain produced activators of cell growth and proliferation, was therefore a possible mechanism by which cells maintain the senescent phenotype (35).

Vinorelbine alone or combined with 5-Fluorouracil has also been shown to induce senescence when given in low doses in a metronomic schedule. Cerrito and colleagues demonstrated this through showing an increase in SA-β-gal positive MDA-MB-231 cells, in the Victor-0 study. The study found that autophagy and cellular senescence contribute more to growth suppression following metronomic therapy than apoptosis (45). The authors conclude that these molecular mechanisms may underlie the positive clinical outcomes seen in the Victor-2 study, using metronomic chemotherapy, with Vinorelbine used in combination with Capecitabine (46).

#### Targeted therapy

Besides chemotherapy, PARP inhibitors have also been shown to induce senescence in TNBC. Fleury and colleagues demonstrated that MDA-MB-231 cells enter senescence after escalating dose of Olaparib. Beyond an increase in SA-β-gal positive cells, cell enlargement, G_2_–M phase arrest, and increased expression of Chk2, p21, BCL-XL, IL6, and IL8 was present (7) The presence of γH2AX, a sign of DNA damage, was also seen in these cells after Olaparib treatment. The authors propose a model which outlines that an intermediate level of DNA damage following Olaparib treatment would cause a senescence-like phenotype in cancer cells, with high levels leading to cell death. These senescent cells may then be targeted in combination with senolytics. They demonstrate that a targetable senescence-like phenotype is induced when MDA-MB-231 cells are treated with Olaparib (7).

#### Cyclin-dependent kinase 4/6 inhibitors and aurora kinase inhibitors (AURKi)

Despite the limited efficacy of cyclin-dependent kinase 4/6 (CDK4/6) inhibitors in TNBC compared with ER-positive tumors in clinic, cell lines, such as 4T1, underwent senescence at 5 μmol/L of Palbociclib alongside a progressive decreased level of phosphorylated Rb and increased Bcl-xL (47). Similar induction was also observed with Abemaciclib, with an additional link to increased antitumor immunity (48).

On the other hand, AURKi seem particularly promising in preclinical setting, for several reasons. Two of the three Aurora kinase family members, Aurora-A (AURKA) and B (AURKB), are essential to regulate cell division and in cancer and work as oncogenes for solid malignancies. In breast cancer, AURKA is crucial to stem-cell renewal, and elevated expression is a survival predictor in ER-alfa positive tumors (27). This is in contrast to TNBC, where high expression of Aurora-A is associated with a high proliferation rate, recurrence, and poor survival outcomes (49).

AURK inhibitors are small molecules showing activity against TNBC in the preclinical setting. Inhibition of AURKA by VX-680 decreased cell migration and inhibited proliferation across several cell lines (49). Similarly, across 29 breast cancer cells exposed to ENMD-2076, a dual Aurora and angiogenesis-involved kinase inhibitor with higher selectivity to AURKA, showed significant growth inhibition and senescence induction activity across TNBC cell lines at lower concentrations as opposed to ER or Her2-positive cell lines. The drug also significantly produced tumor growth inhibition in the xenograft models of MDA-MB-468 and MDA-MB-231 derived cells (50). Interestingly, p53 mutation and increased expression was found to be predictors of higher cytotoxicity and pro-apoptotic stimuli (50, 51).

A combination of AURK inhibitors is also effective. MK- 0457, a pan-AURK inhibitor, with Vorinostat, a histone deacetylase inhibitor, showed higher tumor suppression and prolonged MDA-MD-231 xenograft survival. Mechanistically, MK-0457 induced apoptosis by depleting AURK levels and activity, and Vorinostat induced inhibition of the chaperone association of HSP90 with AURKs by inducing its acetylation (52). The administration of the microtubule inhibitor Eribulin to TNBC cell lines and xenografts increased the active form of AURKA. Alisertib (MLN8237), a selective AURKA inhibitor given in monotherapy, suppressed cell proliferation and migration. The combination increased apoptosis and cytotoxic autophagy in metastasis (53). Another interesting synergism with Alisertib is an alfa-PD-L1 antibody. Although alisertib can reshape the microenvironment by inducing a tumor suppressive stimulus, the combination with the PD-L1 agent induced higher and prolonged cell proliferation suppression and levels of interferon-gamma, TNFα, Cd3^+^, and CD8^+^ T cells (54).

## Potential Therapeutic Strategies

### Chemotherapy

Widely available and nonspecific senolysis is unusual but expected to occur in higher concentrations of most anticancer therapy, also associated with increased toxicity (55). However, senescence induction is commonly seen at tolerated doses (Table 1), being valuable for the one-two punch strategy as shown by Table 2. Moreover, the senolytic power of recent target being druggable in TNBC (e.g., Trop-2) and mechanism of chemotherapy drug delivery (e.g., antibody–drug conjugate) require further investigation (56).

### Src inhibitors

The Src family includes 11 members, such as c-Src, which are a family of non-receptor tyrosine kinases. Src is known to be involved in cell proliferation, invasion, and metastasis in breast cancer, among other biological processes (57). It can be activated by signal transduction from several membrane receptor ligands, including the abundant EGFR receptors in TNBC (58). Moreover, nearly all TNBCs show cytoplasmic expression of Src and 4/5 have membranous expression (59). Membranous expression of Src is currently known as a predictor of Src inhibitor's activity when aberrant (60).

Across the available Src inhibitors that have shown activity against breast cancer, dasatinib accounts for most of the preclinical and clinical evidence in TNBC. Beyond Src, the orally bioavailable tyrosine kinase inhibitor exercises activity against other kinases such as the BCR-ABL, PDGFRB, and Kit (60).

Dasatinib shows activity as monotherapy, and a synergistic effect is reported in combination with chemotherapy in TNBC cell lines (59). It restores Paclitaxel sensitivity in TNBC-resistant cell lineages and reduces the number of stem cells. Their viability, which was also validated in xenografts, included some subjects who achieved complete response (61). On the other hand, in another study where paclitaxel-resistant MDA-MB-231 cells, six days of treatment with Dasatinib did not revert resistance, and only Doxorubicin was able to reduce cell viability (62). Intriguingly, on both Hs578T and MDA-MB-231 cell lines, Dasatinib promoted attenuation of AKT/STAT3 phosphorylation and enhanced the sensitivity to Doxorubicin (63)

Dasatinib also shows synergism with agents targeting DNA-repair pathway. When combined with Cisplatin, Dasatinib drives cell proliferation inhibition (59). The combination with Veliparib and Carboplatin in xenograft models promoted a more significant decrease of the tumor burden than single or double combinations. On this study, the treatment with Veliparib and carboplatin was found to increase Src phosphorylation (64). The combination with Olaparib has also proven synergistic, regardless of DNA-damage repair signature or BRCA mutation (65). We believe these data are highly relevant given the current therapeutic arsenal available in the clinic for this subset of tumors (66).

Although Dasatinib inhibition of Src activation is expected in monotherapy, it also inhibits AKT phosphorylation in TNBC cell lines, suggesting that exploring target therapy might be strategic. Dasatinib combination with an AKT (MK2206) or mTOR (Rapamycin) inhibitor suppressed the proliferation of Dasatinib-resistance cells (67). Anti-EGFR (Cetuximab) plus chemotherapy (Cisplatin) also synergized with Dasatinib. Although Dasatinib reduced cell migration and invasion in monotherapy, it also combined with the other two agents, showing synergism in 6/7 cell lines (68).

Higher cytotoxicity in cell lines was also reported when dasatinib was combined with Afatinib (EGRF inhibitor) and Trametinib (MEK inhibitor). In this study, 33 drugs were evaluated into 12 groups in TNBC cell lines using a combination discovery approach. Five groups were followed up mTOR inhibitors, EGFR inhibitors, BCL inhibitors, MEK inhibitors, and a Super Group B (PI3Kδ inhibitor, JAK inhibitors, BTK inhibitor; ref. 69). Afatinib in combination with Dasatinib was also reported to be effective against 13/14 TNBC cell lines in another study. Interestingly, the authors reported that sensitivity to Afatinib was associated with high baseline levels of pSrc(Y416) and pMAPK(p38), but the combination showed synergistic action when low levels of Bcl2 and mTOR were present (70). Finally, dasatinib also works as a sensitizing c-met inhibitor agent (71).

Beyond the synergism with dasatinib, several of drugs mentioned above are reported to suppress the SASPs, for instance JAK and mTOR inhibitors and flavonoids which will be explored on the next section (72).

Dasatinib monotherapy is effective in TNBC preclinical studies; whereas translation to the clinical setting showed modest activity (Table 3). In a monotherapy phase II study, dasatinib achieved a chemical-biological-radiological (CBR) response of 9.3% (4/43), 2 PR (partial response), and 2 SD (stable disease). SAEs were 13%/48% of respectively 70 mg/100 mg dose, Grade 3 toxicity included 10%/26% GI, 4%/9% pleural effusion, 0/9% generalized edema or pericardial effusions (73). A phase II Dasatinib 100 mg once daily in a neoadjuvant scenario for TNBC 22 recruited patients, of which 2 exhibited PR, 15 SD, and 5PD, was terminated due to futility after an interim analysis (NCT00817531).

**Table 3.** Examples of senolytics clinical performance in solid tumors and breast cancer.

Molecule	Phase	Population	Drug administration	Main results
Dasatinib monotherapy (73)	2	44 patients with TNBC	100 mg twice a day then 70 mg twice a day	PR = 2 pts, SD = 11 pts, CBR = 9.3%. No G4 events amongst the doses but G3 events included diarrhea (10–26%), oedema and effusions (0–9% each).
Dasatinib + ixabepilone (149)	1	19 patients (6 with breast cancer)	MTD: dasatinib 100 mg and ixabepilone 40 mg/m^2^	SD = 1pt, SD = 12pts (2 had breast cancer). G 3/4 toxicities included anemia (37%), fatigue (32%), neutropenia (21%), diarrhea (16%) and G3 QTC prolongation in 1 pt.
Dasatinib + paclitaxel (76)	2	40 patients with breast cancer (8 with TNBC)	Paclitaxel 80 mg (weekly) and dasatinib 120 mg daily	CR = 1 pt (TNBC), PR = 7 pts, ORR = 23%, CBR: 43%. mPFS = 5.2 months, mOS = 20.6 months, TTF = 5.84 months and 1.8 months for TNBC. Adverse events included fatigue (G1 = 75% and G2 = 10%), neuropathy (G1 = 65% and G2 = 2.5%) and diarrhea (G1 = 50% and G2 = 15%). G 3/4 events included neutropenia and anemia (10% each), Lymphopenia (20%), hyperphosphatemia and hyperglycemia (5% each), Thrombocytopenia, Hypokalemia and ALT elevation (2.5% each)
Dasatinib + paclitaxel (75)	1	15 (6 with TNBC)	MTD: dasatinib 120 mg and paclitaxel was given at 80 mg/mg weekly (3 on 1 off) through the escalation period	PR: = 4 pts (two previously treated with paclitaxel) and SD = 5 pts. Edema in 4 pts (G1 = 3 and G2 = 1), majority of other effects were G1/2 and include diarrhea, infection, QTc prolongation. G3 events included neutropenia (2 pts), hyperkalemia (1 pt), fatigue (3 pts), neuropathy (1 pt)
Dasatinib + capecitabine (77)	1	52 (16 with TNBC)	MTD: dasatinib 100 mg/d and capecitabine 1,000 mg/m^2^ twice a day from days 1–14	mPFS = 14.4 weeks (HR+ = 23.1 and TNBC = 7.7 weeks), PR: 24% (TNBC 1/6 pts and HR+ 8/23 pts). G3/4 toxicity at expansion cohort included hand-foot syndrome (10%) and pleural effusion (10%), vomiting, rash, and mucositis (6.7% each), diarrhea, fatigue, dyspnoa, headache, and pain (3.3% each). Decreases in plasma VEGF-A and increases in VEGFR-2 were reported
Bosutinib (79)	2	73 (13 with TNBC)	400 mg/day	PFS rate at 16 weeks: 39.6% (TNBC: 25%); CBR: 27.4% (TNBC: 7.7%). All responding patients were HR+ (4 pts). Most common G3/4 LFT elevation (19%), diarrhea (6%), fatigue (4%), asthenia (6%), infections (3%), and back pain (4%)
Bosutinib + capecitabine (150)	1	32 (11 with breast cancer)	MTD: bosutinib 300 mg daily and capecitabine 1,000 mg/m^2^ twice daily day 1–14	PR = 2 pt (BC = 1) and SD lasting ≤24 weeks = 10 pts (BC = 3) and SD >24 weeks = 4 pt (BC = 2). Most common G3/4 events were mucosal inflammation, hand-foot syndrome, increased AST (9% each), diarrhea, and fatigue (6% each)
AZD0530 (saracatinib; ref. 151)	1	12 (1 with breast cancer)	MTD: 125 mg	SD ≥ 6 weeks = 27%. Most common adverse event was diarrhea (67%), G 3/4 toxicities were lymphopenia, leukopenia, neutropenia, anemia (17% each), and febrile neutropenia, AST increase (8% each)
AZD0530 (152)	1	81 (11 with breast cancer)	RP2D: 125 mg	Confirmed SD = 11 pt. Grade 3/4 toxicities included fatigue and asthenia (3.7% each), anaemia and neutropenia (2.5% each).
AZD0530 + paclitaxel and/or carboplatin (153)	1	116 (7 with breast cancer)	acceptable toxicity of saracatinib at 175mg combined with paclitaxel (175 mg/m^2^ Three-weekly or 80 mg/m^2^ Weekly) with or without carboplatin AUC 5	ORR Carboplatin + Paclitaxel+AZD0530 arm: 11% (1 BC) and on paclitaxel+AZD0530 arm: 21%. Grade 3/4 toxicities were 10–33% amongst dose levels. The most frequent on first cycle included neutropenia, neutropenic sepsis, febrile neutropenia, fatigue, ulcerative colitis, and hyponatraemia
AZD0530 + cediranib (AZD2171; ref. 154)	1	39 (1 with breast cancer)	saracatinib 175 mg/day was better tolerated with Cediranib 20 or 30 mg/day	SD ≥ 8 weeks = 22/35 evaluable pts and 12/35 experienced tumor shrinkage. G 3/4 toxicities included diarrhoea and fatigue (12.8% each), thrombocytopenia (7.7%), anorexia, hypertension, and proteinuria (5.1% each)
Navitoclax + paclitaxel ± carboplatin (109)	1	19 (1 with breast cancer)	Stopped the de-escalation given the significant toxicity	SD = 7/19 pts and PR = 1/19 pts. mPFS = 46 days and mOS: = 236 days. Grade 3/4 toxicities included neutropenia and thrombocytopenia (36.8% each), anemia (26.3%), and fatigue (10.5%)
Navitoclax + docetaxel (155)	1	41 (5 with breast cancer)	MDT: navitoclax 150 mg on days 1–5 and docetaxel 75 mg/m^2^ on day 1	PR = 4/35 evaluable patients. Grade 3/4 events included neutropenia (49%), thrombocytopenia (34%), febrile neutropenia (27%), leukopenia (24%), fatigue (17%), and hyponatremia (15%)
Navitoclax + gemcitabine (110)	1	46 (1 with breast cancer)	MDT: navitoclax 325 mg on days 1–3, 8–10, and 15–17 and gemcitabine 1,000 mg/m^2^ on days 1, 8, and 15	SD rate at end of Cycle 2 = 54% and mPFS = 1.6 months. Main grade 3/4 events included thrombocytopenia (39.1%), neutropenia (19.6%), diarrhea (13%), and anemia (8.7%)
Navitoclax + trametinib (156)	1	43	RP2D: trametinib 2 mg on days 1–14 and navitoclax 25 mg on days 1–28.	Grade 3/4 toxicities occurred in 40%, the most common being AST increase, diarrhea, and thrombocytopenia. At RP2D level, PR = 15.4%, DCR = 46.2%
AT-101 + paclitaxel and carboplatin (157)	1	24 (3 with breast cancer)	AT-101 40 mg twice daily on days 1–3 was tolerable alongside the chemotherapy	CR = 1/24 pts and PR = 4/24 pts. Grade 3/4 toxicities were rare and included abdominal pain, metabolism disorders, elevated LFTs, fatigue, and constipation
ABBV-075 (mirabresib) (158)	1	72 (8 with breast cancer)	RP2D: 1.5 mg daily, 2.5 mg (4 days on 3 off) and 3 mg (Monday/Wednesday/Friday)	SD = 25/65 available pts and mPFS = 1.8 months. Grade 3/4 toxicities occurred in 72.2% the most common being thrombocytopenia (30.6%) and anemia (15.3%)

Abbreviations: AST, aspartate aminotransferase; CBR, clinical benefit rate; CR, complete response; DCR, disease control rate; HR, hormonal receptor; LFT, liver function test; mOS, median overall survival; mPFS, median progression-free survival; PFS, progression-free survival; PR, partial response; PT, patient; QTC, corrected QT interval; RP2D, recommended Phase 2 dose; SD, stable disease; TNBC, triple-negative breast cancer; TTF, time to treatment failure; VEGF, vascular endothelial growth factor.

Given its efficacy in monotherapy, signatures predicting dasatinib benefit would be useful for better selection of patients. This was evaluated by a clinical trial that recruited 97 patients where 93 received a biopsy, and 80% of those were evaluable. Unfortunately, based on predictive gene signatures, the three arms (cell line–derived Dasatinib predictor arm, SRC pathway activity arm Dasatinib, and target index arm) failed to elect patients likely to respond and were closed early. Only one patient experienced stable disease for 300 days (74).

On the other hand, the combinations were associated with more activity. In a phase I dose-escalation alongside Paclitaxel, 6 of 14 patients were triple negative; of the available patients (*N* = 13), 31% achieved PR and 29% SD, although the authors did not disclose the percentage of TNBC on those (75). In a small phase II trial combining Paclitaxel and Dasatinib 120 mg/day stopped by slow accrual, 8 of 40 of recruited patients were triple negative, and only one had clinical benefit. On the one hand, the ORR, mPFS, and mOS were respectively 23%, 5.2 (95% CI, 2.9–9.9), and 20.6 (95% CI, 12.9–25.2) months for the whole group, 3 of 35 of available subjects showed disease response. There was one complete response associated with TNBC, suggesting that the synergistic action might occur in the clinical setting (76).

Other combinations included Capecitabine in a phase 1 trial of dose escalation and expansion, where 16/52 were TNBC. The authors reported 25 response-evaluable patients treated at dose level 3a (Capecitabine 1000 mg/m2 TDS and dasatinib 100 mg day), PR 24% and SD 32%; mPFS 14.4 weeks (77). During a Phase 1/2 of Ixabepilone on day 1, 8, 15 on a 28 days cycle alongside Dasatinib continuously daily. Phase 1 DLT grade 2 leukopenia and thrombocytopenia at dasatinib 140 mg and Ixabepilone 20 mg/m2 (78). Other combinations for which the results are not available yet include Gemcitabine (NCT02720185), Bevacizumab, and Paclitaxel (NCT01015222).

Other Src inhibitors also have shown modest activity against TNBC in monotherapy. Bosutinib, an ARC/ABL tyrosine kinase inhibitor orally bioavailable was evaluated in a phase II trial where 13/73 patients had metastatic TNBC. The PFS at 16 weeks was 25.0% (95% CI, 6.0%–50.5%). From ITT triple-negative population 9/13 PD, 3/13 experienced SD, 1/13 > 24 weeks, CBR 7.7% (0.2%–36%) and from evaluable population 3/11 experienced SD, 1/13 >24 weeks, CBR 9.1% (0.2%–41.3%), 8/13 PD (79). Saracatinib (AZD0530; AstraZeneca), an oral TKI selective for Src was evaluated in a small phase II study with TNBC. 3/9 showed SD <6 months and a medial time to failure of 82 days. The authors concluded that there was no significant single-agent activity to suggest continuing the study (80).

Finally, based on the evidence discussed before, we believe Dasatinib could be a relevant option as a senolytic agent for treatment-induced senescence in TNBC, as it promotes clearance of senescent cells and synergizes with chemotherapy and targeted therapy in preclinical setting (55, 81).

### Flavonoids

Senolytic activity of dietary flavonoids (e.g., Quercetin and Fisetin) was first reported in endothelial human cell lines. Then, significantly more activity was reported in combination with Dasatinib against mouse embryonic fibroblasts, suggesting limited senolytic power either in the natural form or monotherapy (82).

Therefore, in a preclinical study where three quercetin derivates were synthesized, blocking hydroxy groups aiming to increase the transit among bio membranes resulted in evasion of enzymatic degradation and were evaluated *in vitro* in Etoposide-induced senescent MDA-MB-231 cells. The second derivate, for which Quercetin catechol moiety was transformed into diphenyl methylene ketal and had three acetyl groups added, showed potent senolytic activity with tropism to cancer cells (55). Higher anticancer activity was also observed with a third derivate, methoxylated Quercetin glycoside (83). Restoration of chemosensitivity in MDA-MB-231 cell lines was also shown when rutin, a Quercetin glycoside, was administered alongside cyclophosphamide and methotrexate (84).

Quercetin's mechanism of action seems to include disruption of TNBC cells adhesion and migration by inhibiting HuR protein, which directly impacts on the integrity of β-catenin and CD44 function (85). This was also supported by another publication where quercetin provided antitumor activity alongside Doxorubicin, inhibiting migration of TNBC cell lines by modulating β-catenin on a nuclear level (86). The combination of Quercetin with Curcumin also promotes dose-dependent of BRCA1 modulation in some intrinsic TNBC subtypes and induces BRCA1 promoter histone acetylation. The combination reduced the expression of BRCA 1 in MDA-MB-231 cells that are claudin-low. The combination reduced cell survival and migration on both MDA-MB-468 and MDA-MB-231 cell lines more effectively than each drug alone (81). Quercetin mechanism in MDA-MB-231 cells includes induction at the protein level, transcriptional activity, and nuclear translocation of Foxo3a (87).

The mechanism of action of Fisetin includes reversion of epithelium–mesenchymal transition by suppressing the PTEN/Akt/GSK3β pathway. In MDA-MB-231 cells, Fisetin inhibited cell proliferation, migration and invasion, and growth and metastasis of TNBC *in vivo* (88). Similar results were reported by another study using *in vivo* models and 4T1 cells, where Fisetin induced regulation of PI3K/Akt/mTOR pathway (89).

Clinical data on flavonoids are very limited, and the scarce evidence suggests a very modest if any activity in monotherapy in solid tumors (90).

### BH3 mimetics

Because senescence is a response to unrepairable damage, senescent cells must upregulate prosurvival pathways to remain alive. One example is that a hallmark of senescence is the overexpression of anti-apoptotic factors, such as the BCL-2 family of proteins. The complex activation of the proteins can trigger cell death by inducing mitochondria permeabilization leading to caspase activation resulting in apoptosis, which can be otherwise halted in case of anti-apoptotic stimuli (91). Among one of the most studied drug classes to target senescent cells, the BH3 mimetics are a collection of compounds targeting different proteins, either pro (BAX and BAK) or anti-apoptotic (BCL-2, BLC-XL, MCL-1), from the BCL-2 family.

An evolution of the previous analogue ABT-737, Navitoclax (ABT-263) is an orally bioavailable drug that inhibits BCL-2, BCL-XL, and BCL-W, therefore inducing apoptosis. In TNBC, the drug shows mild monotherapy activity with best outcomes observed concomitantly or sequentially. For instance, on Paclitaxel-resistant MDA-MB-231 cells, desensitization to Birinapant BV6, a mitochondria-derived activator of caspase (SMAC) mimetic, was observed but not to ABT-263 (92).

Targeted therapies also added an anti-proliferative effect when combined with Navitoclax. The drug-induced apoptosis in MDA-MB-231 cell lines, but not in MCF-7, was then sensible to the combination with the mTOR inhibitor Everolimus (41). When other mTOR inhibitors, BEX235 or AZD8055, were combined with ABT-263 *in vitro*, a higher rate of apoptosis was observed, and either one of the mTOR combined inhibitors with navitoclax induced more tumor regression in xenograft models than the drugs alone (93). The PI3K and mTOR inhibitor (NVP-BEZ235) and ABT-263 combination, even at lower concentrations, also showed strong synergism against breast cancer cells, especially in ER- and PR-negative cells (94). Induction of BCL-family activity is expected by the mTOR inhibitors, which could sensitize the cell to ABT-263 (95).

The ALK/ROS1 inhibitor Crizotinib induced a significant antiproliferative effect in combination with ABT-263 and a pro-apoptotic stimulus in basal and mesenchymal stem cell-like subtypes (39). Moreover, Navitoclax treatment breaks are associated with early MDA-MB-231 cell growth, but this does not occur in lower doses of Navitoclax + Crizotinib or Crizotinib alone (96). Finally, the senolytic activity of ABT-263 was demonstrated following senescence induction by Alisertib in MDA-MB-231 cell lines (97).

Navitoclax also exhibited synergistic action with other senescence-related mitochondrial proteins. When combined with an MCL-1 downregulator Acriflavine, showing increased toxic activity in MDA- MB-231 and HS578T cell lines, dose, and time-dependent (98). A-1210477, a direct MCL-1 inhibitor or the indirect inhibitor Flavopiridol, synergistically inhibited the growth of HCC-1806 cell line alongside Navitoclax. These suggest that targeting two proteins of the BCL family, in this case, BCL-XL and MCL-1, might induce more activity (99). A synergistic combination with ABT-263 is with the mitochondrial matrix chaperone inhibitor (HSP90 inhibitor) Gamitrinib G-TPP. In the preclinical *in vitro* and *in vivo* study of several tumor types, including TNBC, massive activation of apoptosis was observed (100).

Other synergistic combinations with ABT-263 include Paclitaxel, XL-184 (VEGFR2/MET inhibitor; ref. 39), ATF5 peptide (101), ABT-414, and ABBV-321 (EGFR antibody–drug conjugates; ref. 102), but as expected, there are resistance mechanisms (41). In exposed MDA-MB-231 cell lines, 2,350 upregulated genes were found to be related to resistance and 18-gene resistance signature proposed. After screening for potential options to overcome, reported Tozasertib (AURK-inhibitor) and NU7441 (DNA-dependent protein kinase inhibitor) among the drug tested, able to promote cell toxicity (103).

There are several other BCL-2/BCL-XL inhibitors available, but limited evidence in TNBC. For instance, Sabutoclax overcame resistance in multidrug-resistant breast cancer cell lines (104). Obatoclax showed similar apoptotic activity compared with navitoclax in combination with Gamitrinib-TPP in basal cell lines (100); ABT-737 in combination with GO-203 (MUC1-C inhibitor) inhibited proliferation of TNBC cell lines resistant to ABT-737 and ABT-263 (105); and Venetoclax (ABT-199) showed synergism with Doxorubicin (106) and with Cisplatin (107).

In monotherapy, Navitoclax showed clinical benefit in small cell lung cancers, but limited in breast cancer subtypes (Table 3). The main toxicity includes gastrointestinal (40% diarrhea, 34% nausea, and 36% vomiting), fatigue 34% and dose schedule-dependent thrombocytopenia 14% (108). In a phase I dose escalation study of Navitoclax combination with Carboplatin and Paclitaxel or Paclitaxel, hematologic toxicities including anemia, neutropenia, and thrombocytopenia were listed as G3/4. Although 10/19 patients experienced an SAE, only 1 patient experienced a partial response, and 7 experienced stable disease generating a median PFS of 46 days (109). The combination with Gemcitabine was less toxic in a dose escalation study of 46 patients, the MDT of Navitoclax 325 mg (D1–3 and D8–10) + Gemcitabine (D1 and D8) 1,000 mg rates of G3/4 neutropenia (33%), thrombocytopenia (40%), and anemia (16%). The best response was stable disease for 54% of 39 evaluable patients, and no objective response was observed (110).

Clinical experience with BCL inhibitors was significantly impacted by toxicity, which limited dose escalation in combination with chemotherapy and low efficacy in monotherapy (Table 3). On the other hand, new generation BCL2 inhibitors might offer more safety, indirectly impacting efficacy. For instance, Palcitoclax (APG1252) showed more encouraging data in monotherapy in a dose escalation study of 42 patients. Eight escalating doses of the drug were given either once or twice a week to several patients, and 240 mg once weekly was defined as recommended phase II dose. Five of the 36 evaluable patients experienced a PR and 8 SD (111).

### Bromodomain and extra terminal domain inhibitors

Bromodomain and extra terminal domain (BET) is a class of epigenetic targets composed of four proteins: Brd2, Brd3, Brd4, and bromodomain testis-specific protein (BRDT). The BET proteins have multiple mechanisms of action, the two best studied of which are transcriptional and cell-cycle regulation. They are often dysfunctional in cancer and recently were found to be a potential therapeutic target (112).

The available BET inhibitors have shown preclinical activity in TNBC breast cancer cells in monotherapy. For instance, JQ1, a small molecule that inhibits both BRD4 and c-MYC, was evaluated across intrinsic subtypes of TNBC cell lines promoted dose depended on apoptosis and senescence, both *in vivo* and *in vitro*. In the same study, another two BET-inhibitors showed similar performance I-BET151 and I-BET762 (40, 113).

A combination of BET inhibitors showed encouraging results. In monotherapy or combination, four breast cancer cell lines, three TN, were exposed to JQ1 and a RAC1 inhibitor (NSC23766). Both drugs induced autophagy and suppressed growth alone but more pronouncedly in combination, which was also observed in xenograft models (114).

In another study, the authors performed a library targeting screen of 175 compounds with BET inhibitor JQ1 and OTX015, another BET inhibitor, across six lines of TNBC identified MEK inhibitors (Trametinib and Selumetinib), ERK inhibitor (SCH772984), and AURKIs (Alisertib and Barasertib) among the most synergistic. The most expressive was the p21-activated kinase inhibitor PF-3758309 and a novel synergistic effect between the inhibitor of bromodomain BAZ2/BRD9 with JQ1 (115).

On the other hand, resistance can be expected from combinations with BBI. Although JQ1 combined with Palbociclib or paclitaxel synergistically inhibited the growth of TNBC cell lines, the sequence of administration of the drugs was important to the effect, and senescence played a relevant role in the resistance for all combinations. Interestingly, BCL2L1 and MYC were particularly highly expressed in resistance to the JQ1+Palbociclib compared with JQ1 alone (116). However, very encouraging data from a group based in Ohio suggest that not just Bcl-xL levels could be predictors of BET-inhibitor response but also predict synergism with BH3 mimetics. Their analyses conducted among TNBC cell lines, senescent after the exposure to JQ1, showed that Obatoclax could induce senolysis, and the combination of the two compounds was statistically significant synergistic (117). Similarly, ABT-737, a Bcl-xL inhibitor, was also reported potentially overcome resistance to BET inhibitors by another group, finding CK2 inhibitors as an option for BBI resistance (113).

Clinical data of BET inhibitors are highly awaited in the TNBC population once several compounds evaluated in solid tumors are transitioning from phase 1, such as AZD5153 (118), Birabresib (MK-8628/OTX015; ref. 119), PLX51107 (120), and BMS-986158 (ref. 121; Table 3).

## Challenges and Future Perspectives

### Noninvasive tools to evaluate senescence

One of the most significant challenges in targeting senescence is to uniformize detection, yet noninvasive, sensitive, and accurate detection of senescent cells will be an invaluable and essential tool in the clinical setting: First of all, to allow detection of patients responding positively to prosenescent therapy (e.g., chemo- or radiotherapy), second to enable assessment of the tumor burden posttherapy longitudinally, third to validate efficacy of potential senotherapy and finally, as a potential prognostic tool for patients in the context of minimal residual disease. The burden of senescent cells does not always reflect the degree of aggressiveness of a tumor, therefore sensitivity of the probe is a key asset.

As described in previous sections, there is no single marker of senescence, and it is identified via a variety of immunohistochemical approaches *in vitro*. Sensitive and specific senescence-detection tools need to be assessed and validated *in vivo* and in the human setting. Of all the various markers of senescence, β-Gal activity is an attractive contender. It can be simply and accurately monitored using commercially available chromofluorogenic molecular-based probes, however barriers to *in vivo* application include toxicity of the probes and the requirement of *a priori* formalin fixation of cells (122). Furthermore, it is not an unequivocal biomarker. For instance, MDA-MB-231 cells exposed to paclitaxel, 5-FU and oxaliplatin induced senescence did not significantly increase SA-β-gal positivity when compared with other chemotherapy agents such as doxorubicin and irinotecan. The following are examples of ongoing strategies to enable real-time monitoring of senescence being developed to overcome these challenges:

#### Fluorescent senoprobes

Lozano-Torres and colleagues have investigated several napthalimide-based two-photon probes, which give rise to a detectable “on-off” signal. One such probe (AHGa) contains a naphtalimide core, an I-histidine methyl-ester linker and an acetylated galactose bonded to one of the aromatic nitrogen atoms of the I-histidine through a hydrolysable N-glycosidic bond. On entry into senescent cells, AHGa is transformed into AH, which results in enhanced fluorescence. This was validated in SK-MEL-103 (human melanoma) cell line undergoing Palbociclib-induced senescence (123). Lozano-Torres and colleagues developed a further napthalimide-based two-photon probe (HeckGal) for the detection of cellular senescence both *in vitro* and *in vivo*. Hydrolyzation of HeckGal via the increased lysosomal β-galactosidase activity of senescent cells results in fluorescence emission. They validated HeckGal *in vivo* in a number of mouse models including an orthotopic breast cancer mouse model treated with senescence-inducing Palbociclib (122).

#### PET detectable probes

For the detection probe to be clinically useful, it needs to be detectable via standard imaging modalities. Krueger and colleagues successfully evaluated the PET tracer FPyGal for noninvasive imaging of β-galactosidase as a surrogate marker for senescence. Following studies in cell lines, mouse models and toxicology studies, they carried out a pilot first-in man study in a patient with cancer with liver metastases. High and heterogeneous tracer uptake was revealed via PET scan in the liver metastases. Preparations are being made to set up clinical trials to include histologic validation (124).

#### MRI probes

Shuo Gao and colleagues developed a novel responsive molecular platform for β-gal activity to be detected via MRI. From studying the literature of colorimetric assays of β-gal, they drew the comparison between the standard Fe^3+^-based MRI contrast agents used in clinical practice and the structure of the intense violet Fe complex, which is formed when well-established β-gal substrate alizarin 2-O-β-d-galactopyranoside (AZ-1) is hydrolyzed and the product aglycone alizarin, chelates with ferric iron (Fe^3+^; ref. 125). Therefore they hypothesized that on delivery and cleavage of the substrate AZ-1 at senescent tumor cells with overexpression of β-gal, the subsequent formation of the Fe complex could produce the ^1^H-MRI effect at areas of β-gal activity. They successfully demonstrated this strategy for detecting β-gal activity with lacZ-transfected human MCf7 breast and PC3 prostate cancer cells by reaction-enhanced ^1^H-MRI T _1_ and T _2_ relaxation mapping (126).

#### Cell-free DNA

Fragmented cell-free DNA (cfDNA) was first discovered by Mandel and Métais (127). It is released into the circulating blood stream and other bodily fluids such as CSF and pleural fluid in both physiologic and pathologic conditions. Given its easy accessibility, it has attracted attention as a potential noninvasive biomarker to identify and monitor abnormalities such as cancer in real time (128). For instance, cfDNA is released by dying cells and therefore an increase in cfDNA may reflect a response to cytotoxic drugs. Rosotrami and colleagues have found that cfDNA kinetics are complex with different patterns of release dependent on the modality of cell death (for instance cell necrosis versus apoptosis), which is influenced by mode of treatment (for instance radiotherapy vs. chemotherapy). Their studies further revealed that cellular senescence inhibits cfDNA release which poses implications for further potential real-time monitoring application (129).

#### Soluble senescence markers

Work carried out on senolytic CAR T cells by Amor and colleagues identified urokinase plasminogen activator receptor (uPAR) as a cell surface protein, which is broadly induced during senescence and can be targeted by uPAR- specific CAR T cells to ablate senescent cells *in vitro* and *in vivo*. They found that both MEK and CD4/6 inhibition therapies induced senescence in KP lung cancer cells; and replication-induced senescence in human primary melanocytes led to an increase in cell-surface uPAR expression and soluble PAR (suPAR). These findings were replicated in patient-derived xenograft (PDX) models of non–small cell lung cancer (NSCLC) treated with senescence-inducing CDK4/6 inhibitors as well as in models of oncogene-induced senescence (via overexpression of transduced Nras^G12V^ or by endogenous Kras^G12D^ expression) in a murine model of senescent pancreatic intraepithelial neoplasia (PanIN). Given that suPAR is secreted into the blood circulation, it presents the possibility of being another easily accessible biomarker (130).

#### Dihomo-prostaglandin released upon senolysis

Wiley and colleagues have found that in addition to the SASP, senescent cells synthesize multiple signaling lipids, known as oxylipins, which are biologically active lipids and produced by oxygenation of polyunsaturated fatty acids (PUFA). Examples include PGD2 and its derivatives, 22 carbon PUFAs, and monosaturated fatty acids (MUFA). Via mass spectrometry of the lipids extracted from mitochondrial dysfunction-associated senescent (MiDAS) cells, they found that certain subsets of lipids increased or decreased significantly upon senescence. They found that the most highly elevated senescence-associated prostaglandin was 1a,1b-dihomo-15-deoxy-Δ12,14-prostaglandin J2 (dihomo-15d-PGJ2), with significant (but less striking) increases noted in other prostaglandin variants. The authors also found elevated concentrations of dihomo-15d-PGJ2 and its biosynthetic enzymes in several cell and tissue types in response to various modes of senescence induction and conclude that released dihomo-15d-PGJ2 can be used as a biomarker for senolysis (131).

### Better models for senescence study and improvement of targeting approach

The heterogeneous characteristics of senescence, not just within the same tumor such as cells that produce SASP and those that do not, raises the hypothesis of likely different phenotypes depending on the tumor or previous therapies. Beyond requiring a deeper knowledge of the current hallmarks of senescence, there is the need to better understand triggers and signaling pathways to produce the next generation of senolytic strategies. Current research from Narita's group, which is aiming to generate an *in vivo* senescence atlas, might help to better delineate what would be the next step to explore (132).

Another challenge is the toxicity and nonspecificity of the available senolytics, which the nanomedicines partly overcome. An activatable prodrug based on navitoclax, Nav-Gal, was validated to be effective against 4T1 senescence-induced cells using Palbociclib by Munoz-Espin Lab. This senescence-specific activatable prodrug could reach an intratumoral concentration in mouse models of cisplatin-induced senescence A549 xenografts (133). Other groups validated the activity of nano-encapsulated navitoclax in orthotopic mouse models with TNBC (47, 134). Nanoparticles loaded with obatoclax showed a response in MDA-MB-231 cells (135), quercetin (136), and when topotecan was conjugated with quercetin (137). Recently published data suggested that dasatinib encapsulation might implicate a higher response due to a reduction in metabolism. Bahman and colleagues generated a micellar dasatinib system using polystyrene co-melic acid (SMA) micelles. When 4T1 cells were treated with free and micellar dasatinib, a significantly more pronounced cytotoxic effect was seen compared with MCF-7 and MDA-MB-231 cells (138). This is bacterial β-gal, so it still remains to be validated in the context of enogenous lysosomal SA-β-gal activity. Other successful encapsulations include magnetically guided micelles with dasatinib enhanced and vincristine plus dasatinib liposomes (139, 140).

Finally, the optimum scenario/schedule of senolytics administration in monotherapy before systemic treatment in noninvasive or early disease, alongside or following senescence inducers, is still a debate. This question was investigated using preclinical models, which are limited in providing direct translation answers, especially given the challenge of reproducing the human complexity, TNBC heterogeneity, and influence of previous therapeutic pressure. However, the data from a new generation of senolytics and combinations with targeted therapies highlighted above are encouraging to our eyes and will hopefully allow quicker translation to the clinic.

## Conclusion Remarks

**Figure 1.**
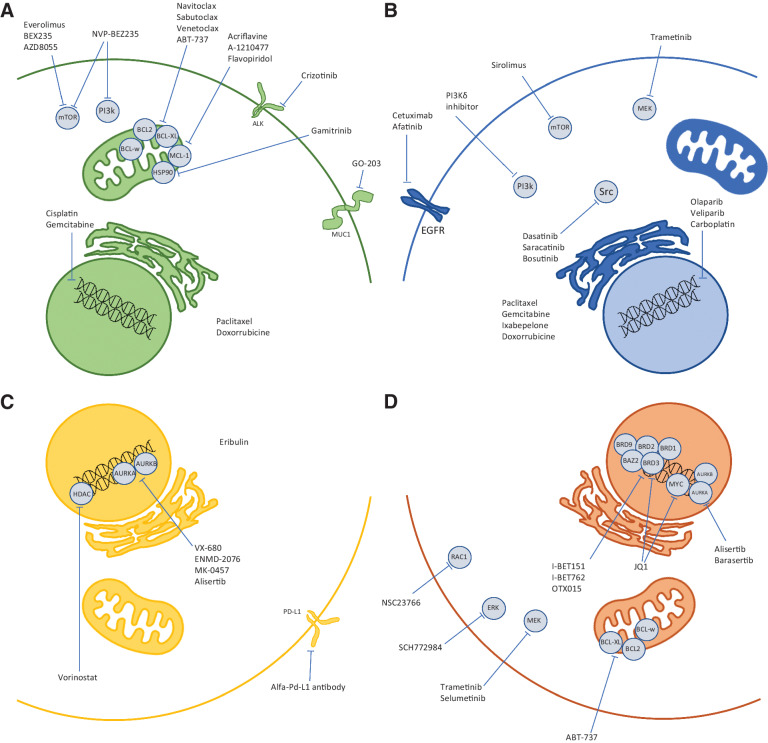
Preclinical data of effective drugs in TNBC that synergize in combination with BH3 mimetics (**A**), Src inhibitors (**B**), AURK inhibitors (**C**), and BET inhibitors (**D**).

**Figure 2.**
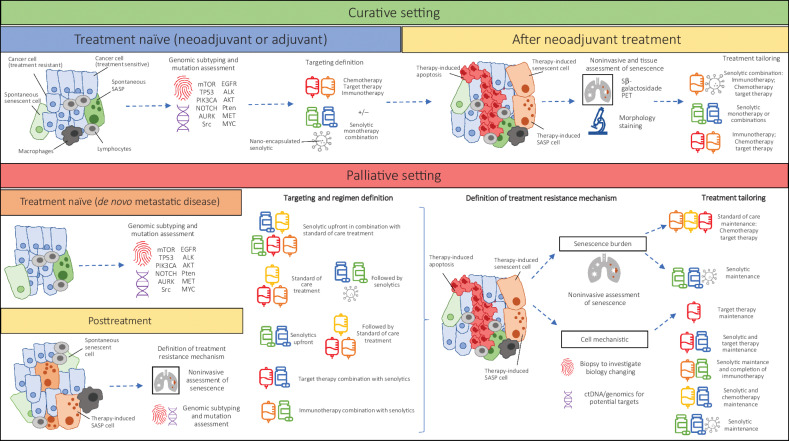
Future-proof options to investigate senescence targeting in TNBC by clinical trials in the curative and palliative settings.

On the basis of the evidence discussed, senescence is emerging as a key driver playing a relevant role in TNBC treatment resistance. Unfortunately, targeting senescence in solid tumor using the previous approaches (e.g., senolytics in monotherapy or combined with chemotherapy) has proven to be limited in effectiveness or associated with intolerable toxicity. Therefore, we hope that the encouraging results of preclinical studies could be translated to clinic using a more targeted approach to induce senescence (e.g., AURK-inhibitors, ADCs, PARP-inhibitors) in combination with next generation of senolytics (e.g., Src-inhibitors, BET-inhibitors, and BH3-mimetics, possibly as nanomedicines; Fig. 1). Moreover, future proof studies might reveal and validate more specific, efficient, and refined options to evaluate senescence burden (e.g., soluble markers, radiologic methods combined with senoprobes) and optimum regimens of senolytics administration (e.g., sequential or concomitant) with current effective anticancer therapies as well as the best window of opportunity (e.g., precursor/noninvasive disease vs. clinically installed malignancy; Fig. 2).
